# Women with IBD Show Higher Psychophysiological Burden in Comparison to Men with IBD

**DOI:** 10.3390/jcm13247806

**Published:** 2024-12-20

**Authors:** Franziska Schulz, Ann Christina Foldenauer, Lara Weidmann, Anne Kerstin Thomann, Andrea Oliver Tal, Sandra Plachta-Danielzik, Thomas Krause, Bernd Bokemeyer, Stefan Zeuzem, Alica Kubesch, Irina Blumenstein

**Affiliations:** 1 Goethe University Frankfurt, Frankfurt University Hospital, Medical Clinic 1, 60596 Frankfurt am Main, Germanystefan.zeuzem@unimedizin-ffm.de (S.Z.); alica.kubesch@unimedizin-ffm.de (A.K.); irina.blumenstein@unimedizin-ffm.de (I.B.); 2 Fraunhofer Institute for Translational Medicine and Pharmacology ITMP, 60596 Frankfurt am Main, Germany; ann.christina.foldenauer@itmp.fraunhofer.de; 3 Department of Medicine II, Medical Faculty Mannheim, Universitätsklinikum Mannheim, Heidelberg University, 68167 Mannheim, Germany; anne.thomann@medma.uni-heidelberg.de; 4 IPG, Medical Practices, 63452 Hanau, Germany; 5 Competence Network IBD, 24103 Kiel, Germany; s.plachta-danielzik@kompetenznetz-darmerkrankungen.de; 6 Gastroenterology Opernstraße, 34117 Kassel, Germany; 7 Interdisciplinary Crohn Colitis Centre, 32429 Minden, Germany

**Keywords:** inflammatory bowel disease, gender-specific differences, psychophysiological impairment, survey

## Abstract

**Background:** Inflammatory bowel disease (IBD) remains an incurable illness. Patients with IBD show gender-specific differences in various aspects of the disease. There is still uncertainty about the causality of the differences. **Methods:** The aim of this study was to determine the most relevant psychophysiological gender-specific differences in IBD. For this purpose, a questionnaire survey was conducted on disease activity and psychological concomitant diseases in patients with IBD (n = 300). Among the 218 patients with IBD who provided gender information, both genders were equally distributed. **Results:** Females with IBD are significantly more affected by IBD-related symptoms than men. Disease activity Scores Harvey-Bradshaw Index (HBI), Partial Mayo Score (pMAYO) showed no significant differences between the sexes in the subgroups with CD (HBI, m: 3 (IQR 1–6), w: 4 (IQR 2–8), *p* = 0.0677) and UC (pMAYO, m: 1 (IQR 0–4), w: 3 (IQR 0–5), *p* = 0.2118). IBD Questionnaire (IBDQ)shows significant differences in the gender-specific analysis. The mean value of the IBDQ total score of the female participants was 4.4 (SD 1.1) and that of men was 5.0 (SD 1.0) (*p* = 0.0002). **Conclusions:** There is a great need to investigate the causality of gender-specific differences in greater depth.

## 1. Introduction

Chronic inflammatory bowel diseases (IBDs) include Crohn’s disease (CD) and ulcerative colitis (UC) [1]. The cause of the inflammation of the gastrointestinal tract that occurs with these diseases is still unknown. Both diseases occur in recurrent episodes and are considered incurable. Due to the chronicity of IBD, it is very difficult to deal with the disease personally, and the likelihood of accompanying psychological complaints is increased. The frequency and prevalence of IBD are constantly increasing [2]. At first glance, patients with IBD hardly differ in terms of their diagnosis, disease phenotype, genetics, and epidemiology. On closer inspection, however, it is noticeable that there are some gender-specific characteristics among those affected [3]. Independent of the underlying disease, women generally seem to experience more stress throughout the course of their lives [4]. This may relate to the social role of women. Gender medicine is gaining increasing attention as a possible key influencing factor in the development and management of chronic inflammatory diseases [5]. This study focused on gender-specific psychophysiological differences in IBD. Special emphasis was placed on the areas of disease perception, disease activity, coping with the disease, and psychological concomitant diseases.

## 2. Materials and Methods

An IBD-specific survey was conducted on the perception of illness, disease activity, coping with the disease, and psychological comorbidities of all participants.

### 2.1. Ethics

This study was approved by the ethics committee of the University Hospital Frankfurt am Main, and each participant signed an electronic informed consent form. Participating patients completed the survey electronically and anonymously; no collection of identifying patient information was created, so no inference of personal data is possible. Ethical committee approval dated 19 August 2020. Vote number: 19–496.

### 2.2. Study Population

The basic population of this study for screening was n = 300. For the present study, all adult patients with a diagnosis of CD and UC confirmed according to ECCO (European Crohn’ s and Colitis Organisation) guideline criteria, who had visited one of the IBD outpatient clinics listed below at least once in the period from November 2020 to November 2021 (12 months) and were willing to participate in the voluntary surveys, were identified. Recruitment was consecutive. The participating outpatient clinics were the IBD Outpatient Clinic of the University Hospital Frankfurt am Main (Prof. Dr. med. Irina Blumenstein), Gastroenterological Practice Minden (Prof. Dr. med. Bernd Bokemeyer), University Outpatient Clinic of the University Hospital Mannheim (PD. Dr. med. Anne Kerstin Thomann), and the Gastroenterological Practice Kassel (Dr. med. Thomas Krause).

The study design consisted of an anonymized survey that was conducted via tablets in the study period from November 2020 to November 2021 (12 months). The survey took place as part of routine visits in the respective IBD outpatient clinics.

### 2.3. Survey and Questionnaires

In addition to questions on demographics, medical history, disease-status-inclusive clinical activity, and psychological health status, the order of the following questionnaires to be completed by the participants was randomized: Disease Activity Scores Harvey-Bradshaw Index (HBI) [6], Partial Mayo Score (pMAYO) [7], IBD questionnaire (IBDQ-32 items) [8], IBD disk [9], Beck’s Depression Index (BDI) [10], Short Health Scale (SHS) [11], Rosenberg Self-Esteem Scale (RSES) [12], Body Image [13], Sexual Health [14], Fatigue (FACIT-F) [15], Work Productivity Index (WPAI) [16], and the Disability Index [17]. The IBDQ and the IBD Disk were examined in particular in this study.

The 32-item IBDQ is a 32-item Likert-based questionnaire divided into four dimensions: bowel symptoms (10 items), systemic symptoms (5 items), emotional function (12 items), and social function (5 items). The responses to each of the questions are graded from 1 (a very severe problem) to 7 (not a problem). The IBDQ item sum provides a possible range of 32 to 224, where a higher score indicates a better quality of life with regard to IBD. IBDQ scores of ≥180 are associated with IBD remission, and patients with scores of ≤130 are associated with severely active IBD [18,19]. The IBDQ subdomain scores are determined as the mean of the associated non-missing item scores of the corresponding subdomain. Similarly, a total IBDQ score can be derived as the total mean of all non-missing IBDQ items. As a result, the total IBDQ and its subdomain scores are also scores with a range of 1 to 7 [8].

The IBD Disk is a questionnaire with 10 items assessing the patient’s state of abdominal pain, defecation, interpersonal interactions, education/work, sleep, energy, emotions, body image, sexual functions, and joint pain in a standardized manner. Each item is scored on a disc-shaped visual analog scale from 0 (absolutely disagree) to 10 (absolutely agree). The results are illustrated in a disk similar to a radar plot to demonstrate the intensity of the patients’ states. In contrast to the IBDQ, the IBD Disk has not been prospectively evaluated [9].

### 2.4. Clinical Activity Scores

Based on the HBI and pMAYO disease activity indices for CD and UC, we derived summarized scores for the stool frequency and general health independent of the IBD type.

### 2.5. Statistical Analyses

All parameters were reported descriptively according to the underlying distribution by mean and standard deviation (SD) or, in the case of skewed distribution, by median and IQR (Q1–Q3, interquartile range). Frequencies are described as the number and percentage of the respective subgroup. The analysis was stratified by gender, whereby only patients who had indicated their gender were included. Comparisons by gender were compared for qualitative endpoints using the Chi-square test or Fisher test (small cell frequencies) and for interval scaled endpoints using Wilcoxon or independent *t*-test for an exploratory significance level of 5% (all tests 2-sided; H0: no difference between sexes). All statistical analyses were performed with SAS 9.4. and EXCEL Version 2108 in MS Office LTSC Professional Plus 2021.

## 3. Results

### 3.1. Survey Response Rate

In total, 300 patients with IBD opened the survey link at least once. A total of 78.3% (235/300) provided partially full answers, and the overall full response rate per question was ~72% (216/300). A total of 225 patients (75%) answered questions on demographic information (e.g., see filled-in weight data). Of the 300 participants, 72.7% (218/300) specified their gender; these 218 participants formed the subgroup analyzed in the following survey results.

### 3.2. Survey Results

Among the 218 patients with IBD who provided gender information, both genders were equally distributed: Demographic data and disease characteristics are displayed in Table 1. A total of 82.9% (92/111) of the men and 85.1% (91/111) of the women stated Germany as their country of birth. The type of IBD was not specified by 20 (18.69%) of the women and 20 (18.02%) of the men. The median time from the onset of the first symptoms to initial diagnosis was 9 months for both female (IQR 3–18.5) and male (IQR 3–19) survey participants. For 43 (40.19%) of the women, it took more than twelve months from the onset of the first IBD symptoms to receive the final diagnosis of IBD. Among the men, 29 (26.13%) had more than twelve months between the first IBD symptoms and the final diagnosis. Compared to five men (4.50%), 18 (16.82%) of the women reported suffering from additional rheumatologic symptoms (rheumatoid arthritis) (*p* = 0.0240). The reported family status was comparable between both genders, while slightly more men had a high school diploma (27.03% men (30/111) vs. 26.17% women (28/111)) or university degree (18.01% men (20/111) vs. 10.28% women (11/111)). Slightly more women stated that they had completed an apprenticeship.

#### 3.2.1. HBI and pMAYO—Clinical Disease Activity

Disease activity (HBI, pMAYO) showed no significant differences between sexes in the subgroups with CD (HBI, m: 3 (IQR 1–6), w: 4 (IQR 2–8), *p* = 0.0677) and UC (pMAYO, m: 1 (IQR 0–4), w: 3 (IQR 0–5), *p* = 0.2118) (see Supplementary Materials, Table S1).

Over 50% of women suffered from additional joint complaints and over 40% from IBD-related skin conditions. Of the male patients with IBD, just over 30% reported both joint and skin complaints. In line with this, almost 30% of male patients with IBD rated their general health as good. Among the female participants, only 20% rated their general health as good at the time of screening. This was equally confirmed by the General Health Score. The number of women who rated their general health as good was only just over 30%, whereas almost 50% of men rated their general health with chronic IBD as good. It is striking that female patients with IBD stated that they were generally more satisfied with themselves despite this. Based on the Rosenberg Self-Esteem Scale (RSES) score, 60.75% of the female study participants confirmed that they were satisfied with themselves; of the men, only 54.05% reported feeling satisfied with themselves

#### 3.2.2. IBDQ

The evaluation of the IBDQ shows significant differences in the gender-specific analysis as well. The mean value of the IBDQ total score of the female participants was 4.4 (SD 1.1) and that of the men was 5.0 (SD 1.0), with *p* = 0.0002 showing that the general IBD-related quality of life was significantly more impaired in women (Table 1). In all four IBDQ sub-dimensions, “social, emotion, systemic, bowel”, there were exploratively significantly worse results for female participants compared with male participants: the mean value for women in the social category was 4.6 (SD 1.5) and for men was 5.3 (SD 1.4) (*p* = 0.0015). The emotion category resulted in a mean value of 4.3 (SD 1.1) for women and 4.6 (SD 1.5) for men (*p* = 0.0001). In the systemic dimension, the mean value for female survey participants was 3.9 (SD 1.1) and 4.4 for males (SD 1.2) (*p* = 0.0017). The last category, bowel, resulted in a mean value of 4.7 (SD 1.3) for women and 5.2 (SD 1.1) for men (*p* = 0.0023) (Table 1).

#### 3.2.3. IBD Disk

**Figure 1:**
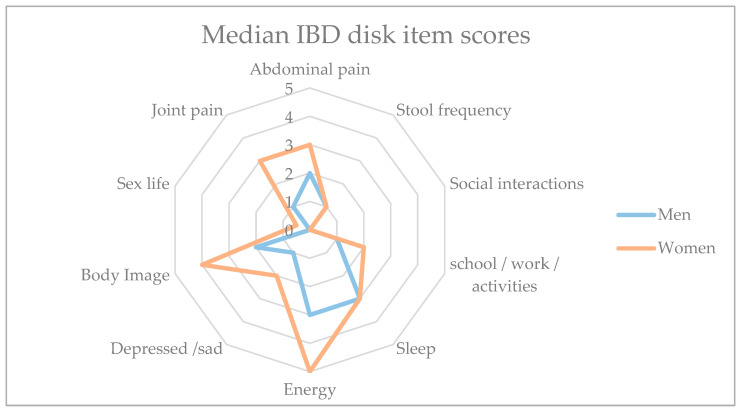
Radar plot of median scores of IBD disk item topics where problems of patients arose (range 0 to 10); higher scores are worse. Women showed overall worse item scores than men in all topics except social interaction. Significantly worse outcomes for female patients with IBD compared with males were observed in the topics of abdominal pain, sleep, energy, depressed/sad state, body image, and joint pain (see Table 1).

The analysis of the IBD disk questionnaire in the abdominal pain category showed that women (3, IQR 1–7) suffered more frequently from abdominal pain than male patients with IBD (2, IQR 1–4) (*p* = 0.0342). Women also suffered more from sleep problems (w: 3 (IQR 1.5–8), m: 3 (IQR 0–7), *p* = 0.0253) than men (Table 1). Women reported having less energy in the last week before testing compared with men (w: 5 (IQR 3–8), m: 3 (IQR 0–7), *p* = 0.0007). Furthermore, female participants were more likely to have an IBD-associated body image issue compared with men with IBD (w: 4 (IQR 2–8), m: 2 (IQR 0–5), *p* = 0.0004) (Figure 1).

#### 3.2.4. Beck’s Depression Index

The Beck’s Depression Index (BDI) total score resulted in a mean value of 11.5 (SD 8.6) for women and a mean value of 7.7 (SD 6.5) for men and is thus significant concerning general well-being in the gender-specific comparison (*p* = 0.0008) (Table 1). A significant difference was found between women and men in the BDI classification. A highly significant number of 39 women (36.45%) stated that they did not suffer from depression associated with IBD, whereas 64 (57.66%) of the male participants confirmed that they had no depression associated with IBD (*p* = 0.0063).

Significantly worse results for women compared with men were also shown in items of the Short Health Scale (SHS) and FACIT-Fatigue scores (m: 91 (IQR 78.4–99.1), w: 83 (IQR 61.0–93.9)), and individual items of the Work Productivity Index (WPAI) questionnaire (Table 1).

## 4. Discussion

To the best of our knowledge, this is the first study that depicts gender-specific psychophysiological differences in IBD in all four relevant categories of bowel, systemic, emotional, and social factors based on the classification of the IBDQ. Women showed a significantly poorer disease-related quality of life in the analysis of the IBDQ despite having comparable disease activity. In all IBDQ sub-dimensions, i.e., social, emotion, systemic, bowel, the disease-related status of female subjects proved to be more complex in terms of the stresses analyzed compared with that of the male study participants.

Taking all the IBDQ sub-dimensions surveyed together, it can be assumed that female IBD patients have a more pronounced holistic disease burden. As a result of the higher disease burden, women suffer more frequently from disease-related sadness and depression. The results of the present study are consistent with the findings of a recently published randomized controlled trial by Bernabeu et al. from 2024, which reports that female patients with IBD suffer significantly more frequently from depression than men with IBD [4].

The higher psychosocial burden of female test subjects is reflected in their perceived quality of life. Accordingly, female IBD patients reported a lower quality of life than the male study participants. As the multicenter study by Chuan et al. from 2023 already shows, our study also shows significantly worse results for women in the quality-of-life category, including sleep quality [20]. In addition, we found a higher level of lack of energy and increased dissatisfaction with their own body image in women with IBD. These factors have a direct impact on both quality of life and psychosocial distress and can be mutually beneficial.

In addition, the present study was able to show that the phase between the onset of the first symptoms and the final diagnosis is longer in female IBD patients than in men. This is confirmed by a study by Sempre et al. from 2023 [21]. The disease-related burden begins for a large number of patients, especially female patients, even before the final diagnosis. This first phase of ignorance, albeit sometimes with severe symptoms, can be an enormous burden for those affected. The longer the phase lasts, the greater the burden at this early stage of the disease.

At the present time, there is still no known causality for the higher burden of disease in female IBD patients. There are assumptions that seek an explanation in the different hormone status of women and men. However, the exact relationship between sex hormones and IBD is still unclear [22].

In the aforementioned study by Chuan et al. 2023, the assumption is made that female IBD patients have a greater need for psychological disease-specific treatment [20]. This finding also corresponds to our view and is supported by the results of the present study. Consequently, the need for an interdisciplinary approach in the treatment and research of patients with IBD is becoming increasingly clear.

Recent studies have reinforced the impression that two components play an essential role with regard to the higher burden of female IBD patients: firstly, which IBD is present; secondly, which phase of the disease the patient is in. It is described that women with Crohn’s disease suffer more from anxiety compared to those with ulcerative colitis [4]. This observation again suggests that the increased psychological stress in women is not purely psychological but, as also described in our work, psychophysiological in origin. The psychophysiological origin of the symptoms does not detract from the focus on the relevance of the psyche of IBD patients described in this paper. On the contrary, in our view, it further illustrates the extent to which psychological and physical symptoms are mutually dependent and result in a symptom construct that can no longer be separated. Regardless of the type of disease, it can be assumed that female IBD patients react more strongly to stressful life events in terms of a reduction in quality of life.

## 5. Limitations of the Study

The present study cannot make any statement about the causality of the differences. Due to the fact that the data were collected in several large IBD centers, objectification is made more difficult by location-dependent study conditions. Furthermore, another study limitation is that our work is a cross-sectional study; thus, a longitudinal study in several centers with a correlation of clinical parameters is required to further confirm our results. This will enable the collection of parameters under investigation at multiple time points. A modified study design would support the necessary advancement toward viewing the findings in the context of the relapsing course of the disease.

Our results and resulting deductions are limited by a potential population bias, as we cannot evaluate if the cohort suffers from an unobserved confounding factor present in the subgroup of patients who denied their participation. Yet, it is notable that with regard to size, the subgroup of our survey within the largest center does reflect a representative subgroup of the IBD patients, with at least 25% of approximately 600 patients (≥150/600) examined during the survey conduction period.

As several data of survey information are missing, bias may arise with regard to the statistical significance and deductions, where at least 10% of the variables is missing (see Table 1 and Section 3.2). Despite the potential bias, the findings did meet our assumptions of the medical assessment of the treating physicians and literature findings, supporting our results and assumptions.

## 6. Conclusions

The results of this study demonstrated that women with IBD are affected by a higher psychosocial symptom burden than men with no difference in clinical disease activity. Interdisciplinary treatment and further research are necessary to determine the cause of the gender-specific differences and their impact on quality of life and to develop appropriate therapeutic approaches.

## Figures and Tables

**Table 1:** Overview table of a selection of IBD survey results of a subgroup with sex specification documented (N = 228). All data are given as number and percentage in the respective sex subgroup (or subgroup thereof), mean (SD), or median (IQR, Q1–Q3) if data distribution was skewed. Statistical tests were performed exploratively at a 5% significance level. BMI: Body Mass Index; IBD: inflammatory bowel disease; IBDQ: Inflammatory Bowel Disease Questionnaire; BDI: Beck’s Depression Index; and SHS: Short Health Scale.

Scheme 111	Parameter Details	Men (N = 111)	Women (N = 107)	*p*-Value Sex Comparison	Test	Significance
Age	Years	39.9 (SD 14.7), Nmiss = 4	37.6 (SD 12.2), Nmiss = 4	*p* = 0.2382	T-test	n.s.
BMI	kg/m^2^	26.2 (SD 6.3), Nmiss = 2	25.7 (SD 6.2), Nmiss = 4	*p* = 0.2504	Wilc	n.s.
IBD type	*Ulcerative Colitis*	42 (37.84%)	31 (28.97%)	M.T.V.	N.t.	/
	*Crohn’s Disease*	49 (44.14%)	56 (52.34%)			
	*Not specified*	20 (18.02%)	20 (18.69%)			
IBDQ score	*Higher scores are better*	5.0 (SD 1.0) Nmiss = 13	4.4 (SD 1.1) Nmiss = 17	*p* = 0.0002	T-test	**
IBDQ score	*Higher scores are better*	5.0 (SD 1.0) Nmiss = 13	4.4 (SD 1.1) Nmiss = 17	*p* = 0.0002	T-test	**
IBDQ item sum	*Higher scores are better*	152.9 (SD 35.6) Nmiss =4	134.3 (SD 37.8) Nmiss = 4	*p* = 0.0003	T-test	**
IBDQ category	IBD remission (IBDQ sum ≥ 180)	27 (24.32%)	13 (12.15%)	*p* = 0.0008	CS, w/o missing information	**
	IBD disease active (130 < IBDQ sum < 180)	54 (48.65%)	41 (38.32%)			
	IBD disease severely active (IBDQ sum ≤ 130)	20 (18.02%)	42 (39.25%)			
	Not applicable	10 (9.01%)	11 (10.28%)			
IBDQ category	IBD remission (IBDQ sum ≥ 180)	27 (24.32%)	13 (12.15%)			
BDI total score	*Higher scores are worse*	7.7 (SD 6.5) Nmiss = 13	11.5 (SD 8.6) Nmiss = 12	*p* = 0.0008	Wilc	**
BDI class	*No depression*	64 (57.66%)	39 (36.45%)	*p* = 0.0063	Fisher, w/o missing information	*
	*Neglectable minimal depression*	16 (14.41%)	25 (23.36%)			
	*Mild depression*	12 (10.81%)	19 (17.76%)			
	*Moderate depression*	6 (5.41%)	8 (7.48%)			
	*Severe depression*	/	4 (3.74%)			
	*Missing*	13 (11.71%)	12 (11.21%)			
IBD disk item: problems sleeping last week	*Higher scores are worse*	3 (IQR 0–7) Nmiss = 12	3 (IQR 1.5–8) Nmiss = 7	*p* = 0.0253	Wilc	*
IBD disk item: not enough energy last week	*Higher scores are worse*	3 (IQR 0–7) Nmiss = 12	5 (IQR 3–8) Nmiss = 7	*p* = 0.0007	Wilc	**
IBD disk item: feeling sad, depressed, frightened, etc., last week	*Higher scores are worse*	1 (IQR 0–4) Nmiss = 12	2 (IQR 1–6) Nmiss = 8	*p* = 0.0024	Wilc	*
IBD disk item: body image last week	*Higher scores are worse*	2 (IQR 0–5) Nmiss = 16	4 (IQR 2–8) Nmiss = 7	*p* = 0.0004	Wilc	**
IBD disk item: sex life—mental or physical stress last week	*Higher scores are worse*	0 (IQR 0–2) Nmiss = 30	0.5 (IQR 0–4.5) Nmiss = 31	*p* = 0.0516	Wilc	*
IBD disk item: joint pain last week	*Higher scores are worse*	1 (IQR 0–4) Nmiss = 12	3 (IQR 1–7.5) Nmiss = 7	*p* = 0.0009	Wilc	**
WPAI	*% overall work impairment due to health*	20% (IQR 10–30%) Nmiss = 61	20% (IQR 10–50%) Nmiss = 57	*p* = 0.0257	Wilc	*
SHS: severity of Symptoms	*Higher scores are worse*	30 (IQR 20–50) Nmiss = 15	40 (IQR 20–60) Nmiss = 12	*p* = 0.0157	Wilc	*
SHS: degree of sorrow based on your IBD	*Higher scores are worse*	40 (IQR 20–70) Nmiss = 14	50 (IQR 30–80) Nmiss = 11	*p* = 0.0275	Wilc	*
Fatigue G2 transformed score (scaled 0–100)	*Higher scores are better*	90.8 (IQR 78.4–99.1) Nmiss = 42	83.0 (IQR 61.0–93.9) Nmiss = 39	*p* = 0.0121	Wilc	*

Statistical tests and additional descriptions are abbreviated as follows: Wilc = Wilcoxon test; CS = Chi-square test; N.t. = not tested; W/o = without; SG = Subgroup; NMiss = number of missing observations (only listed if missing occurred); SD = standard deviation; and IQR = interquartile range. Significance: n.s. = not significant; * = *p* < 0.05; ** = *p* < 0.001.

## Data Availability

The data presented in this study are available on request from the corresponding author. The data are not publicly available due to data protection and ethical regulations.

## Supplementary Materials

The following supporting information can be downloaded at: https://www.mdpi.com/article/10.3390/jcm13247806/s1, Table S1: Additional overview table of extract of IBD survey results of subgroup with sex specification documented (N = 228).
