# Supplementary material for: Women with IBD Show Higher Psychophysiological Burden in Comparison to Men with IBD
Source: J Clin Med. 2024 Dec 20;13(24):7806. doi: 10.3390/jcm13247806 (PMC11728100; doi:10.3390/jcm13247806)
Supplement: Supplementary file 1 [file jcm-13-07806-s001.zip › jcm-3342153-supplementary.pdf]

Supplementary Material:

Women with IBD show higher psychophysiological burden in comparison to men with IBD

Questionnaire details:

- Part A: Free text: What is QoL for you?

- Part B: Personal information (demographics, anamenesis, drinking/eating habits, psyche)

- Part C: Disease Activity Scores (16 questions)

- HBI

- SSCAI

- pMAYO

- Part D: Short Health Scale, RSES (14 questions)

- Part E: Body Image (24 questions)

- Part F: Sexual Health (13 questions)

- Part G: Fatigue (40 questions)

- Part H: Work Productivity (4 questions)

- Part I: Disability Index (6 questions)

- Part J: IBDQ (24 questions)

- Part K: BDI (21 questions)

- Part L: IBD Disk (10 questions)

Remaining table with results

Table S1: Additional overview table of extract of IBD survey results of subgroup with sex specification documented (N=228).

		Men	Women	Sex comparison
		N=111	N=107	Test, p-value
**Time since initial IBD diagnosis to 2021**	years	9 (IQR 3-19)<br>Nmiss=3	9 (IQR 3-18.5)<br>Nmiss=3	Wilc,<br>p =0.9758,
**Duration of suffering from**	0-3 months	20 (18.02%)	21 (19.63%)	CS,
	4-6 months	27 (24.32%)	22 (20.56%)	

		Men	Women	Sex comparison
		N=111	N=107	Test, p-value
IBD symptoms prior to IBD diagnosis	7-12 months	21 (18.92%)	17 (15.89%)	p =0.3228, w/o missings
	>12 months	29 (26.13%)	43 (40.19%)	
	Not specified	14 (12.61%)	4 (3.74%)	
Weight	kg	85.0 (SD 21.4) Nmiss=2	71.2 (SD 18.1) Nmiss=4	Wilc, p< 0.0001
Place of birth	Germany	92 (82.88%)	91 (85.05%)	N.t.
	Other	12 (10.81%)	8 (7.48%)	
	Not specified	7 (6.31%)	8 (7.48%)	
Highest school qualification	Secondary school certificate	17 (15.32%)	18 (16.82%)	N.t.
	Apprenticeship / professional training	24 (21.62%)	37 (34.58%)	
	Highschool diploma	30 (27.03%)	28 (26.17%)	
	Bachelor	10 (9.01%)	7 (6.54%)	
	Master / Diploma / PhD	20 (18.01%)	11 (10.28%)	
	Other	10 (9.01%)	6 (5.61%)	
Family status	Married / in legal partnership	47 (42.34%)	49 (45.79%)	N.t.
	Unmarried, in partnership	23 (20.72%)	23 (21.50%)	
	Unmarried	34 (30.63%)	25 (23.36%)	
	Divorced	3 (2.70%)	8 (7.48%)	
	widowed	1 (0.90%)	/	
	Not specified /other	3 (2.70%)	2 (1.87%)	
Concomitant diseases	Cardiovascular diseases	13 (11.71%) Nmiss =76	13 (12.15%) Nmiss = 61	Fisher, p=0.4738 w/o missings
	Pulmonary diseases	13 (11.71%) Nmiss =76	8 (7.48%) Nmiss = 61	Fisher, p=0.0719 w/o missings
	Neurological diseases	6 (5.41%) Nmiss =76	5 (4.67%) Nmiss = 61	Fisher, p=0.5180 w/o missings
	Rheumatological diseases	5 (4.50%) Nmiss =76	18 (16.82%) Nmiss = 61	Fisher, p=0.0240 w/o missings

		Men	Women	Sex comparison
		**N=111**	**N=107**	**Test, p-value**
	Endokrinological diseases	3 (2.70%)<br>Nmiss =76	8 (7.48%)<br>Nmiss = 61	Fisher,<br>p=0.3345<br>w/o missings
Disability Index				
**IBDQ SOCIAL subdomain score**	*Higher score are better*	5.3 (SD 1.4)<br>Nmiss=11	4.6 (SD 1.5)<br>Nmiss=13	Wilc,<br>P=0.0015
**IBDQ EMOTION subdomain score**	*Higher score are better*	4.9 (SD 0.9)<br>Nmiss=7	4.3 (SD 1.1)<br>Nmiss=9	tTest,<br>P< 0.0001
**IBDQ SYSTEMIC subdomain score**	*Higher score are better*	4.4 (SD 1.2)<br>Nmiss =5	3.9 (SD 1.1)<br>Nmiss=9	tTest<br>p=0.0017
**IBDQ BOWEL subdomain score**	*Higher score are better*	5.2 (SD 1.1)<br>Nmiss =8	4.7 (SD 1.3)<br>Nmiss=10	tTest,<br>p=0.0023
IBD disk item: abdominal pain last week	*Higher scores are worse*	2 (IQR 1-4)<br>Nmiss=14	3 (IQR (1-7)<br>Nmiss=10	Wilc,<br>p=0.0342
IBD disk item: Stool frequency last week	*Higher scores are worse*	1 (IQR 0-4)<br>Nmiss=15	1 (IQR 0-4)<br>Nmiss=12	Wilc,<br>p=0.7929
IBD disk item: problems with social interactions last week	*Higher scores are worse*	0 (IQR 0-3)<br>Nmiss=12	0 (IQR 0-5)<br>Nmiss=8	Wilc,<br>p=0.1418
IBD disk item: Problems with school / work / activities last week	*Higher scores are worse*	1 (IQR 0-4)<br>Nmiss=16	2 (IQR 0-6)<br>Nmiss=10	Wilc,<br>p=0.1073
General Health (derived)	*Higher scores are worse*	0 (IQR 0-1)	1 (IQR 0-1)	Wilc,<br>p=0.0264
pMAYO Score<br>*Only assessed in ulcerative colitis subgroup. N=73 with reported sex, (N=77 in total cohort)*	*Higher scores are worse*	1.0 (IQR 0-4)<br>Nmiss (SG)=2	3.0 (IQR 0-5)	Wilc;<br>p=0.2118
PMayo Classification<br>*Only assessed in ulcerative colitis*	Remission	23 (54.76%)	10 (32.26%)	Fisher,<br>p =0.1848,<br>w/o missings
	Mild disease	12 (28.57%)	9 (29.03%)	
	Moderate disease	4 (9.52%)	8 (25.81%)	

		Men	Women	Sex comparison
		N=111	N=107	Test, p-value
subgroup. N=73 with reported sex, (N=77 in total cohort)	Severe disease	3 (7.14%)	2 (6.45%)	
	Missing	/	2 (6.45%)	
HBI<br>*Assessed in Crohn's disease subgroup. N=105 with reported sex, (N=111 in total cohort)*	*Higher scores are worse*	3.0 (IQR 1-6)<br>Nmiss (SG) = 5	4.0 (IQR 2-8)<br>Nmiss (SG)=4	Wilc; p=0.0677
HBI classification<br>*Assessed in Crohn's disease subgroup. N=105 with reported sex, (N=111 in total cohort)*	Remission	28 (57.14%)	27 (48.21%)	Fisher,<br>p =0.4614, w/o missings
	Mild disease	10 (20.41%)	11 (19.64%)	
	Moderate disease	5 (10.20%)	12 (21.43%)	
	Severe disease	1 (2.04%)	2 (3.57%)	
	Missing	5 (10.20%)	4 (7.14%)	
Is your QoL also impaired when you are in remission	Yes	35 (31.53%)	38 (35.51%)	N.t.
	No	45 (40.54%)	43 (40.19%)	
	Not specified	31 (27.93%)	26 (24.30%)	
